# Recrudescent Wave of A/H1N1pdm09 Influenza Viruses in Winter 2012-2013 in Kashmir, India

**DOI:** 10.1371/currents.outbreaks.f1241c3a2625fc7a81bf25eea81f66e6

**Published:** 2013-09-26

**Authors:** Parvaiz Koul, Umar Khan, Khursheed Bhat, Siddhartha Saha, Shobha Broor, Renu Lal, Mandeep Chadha

**Affiliations:** SheriKashmir Institute of Medical Sciences; Internal & Pulmonary Medicine, SKIMS, Srinagar, J&K; Department of Internal and Pulmonary Medicine, SKIMS, Srinagar; India Influenza Center, CDC, Atlanta, Georgia; Director Inclen Laboratory, Inclen trust International, New Delhi; CDCCDC, ATLANTA; National Institute of Virology, Pune

## Abstract

Some parts of world, including India observed a recrudescent wave of influenza A/H1N1pdm09 in 2012. We undertook a study to examine the circulating influenza strains, their clinical association and antigenic characteristics to understand the recrudescent wave of A/H1N1pdm09 from November 26, 2012 to Feb 28, 2013 in Kashmir, India. Of the 751 patients (545 outpatient and 206 hospitalized) presenting with acute respiratory infection at a tertiary care hospital in Srinagar; 184 (24.5%) tested positive for influenza. Further type and subtype analysis revealed that 106 (58%) were influenza A (H1N1pdm09 =105, H3N2=1) and 78 (42%) were influenza B. The influenza positive cases had a higher frequency of chills, nasal discharge, sore throat, body aches and headache, compared to influenza negative cases. Of the 206 patients hospitalized for pneumonia/acute respiratory distress syndrome or an exacerbation of an underlying lung disease, 34 (16.5%) tested positive for influenza (22 for H1N1pdm09, 11 for influenza B). All influenza-positive patients received oseltamivir and while most patients responded well to antiviral therapy and supportive care, 6 patients (4 with H1N1pdm09 and 2 with influenza B) patients died of progressive respiratory failure and multi-organ dysfunction. Following a period of minimal circulation, H1N1pdm09 re-emerged in Kashmir in 2012-2013, causing serious illness and fatalities. As such the healthcare administrators and policy planners need to be wary and monitor the situation closely.

## Introduction

The 2009 pandemic influenza A virus (A/H1N1pdm09) caused a pandemic in 2009 and was followed by a phase of post-pandemic transmission in 2010 1 ^,^ 2 ^,^ 3 . Although severe illness and deaths were initially reported during the pandemic phase, disease severity was largely comparable to seasonal influenza; illness was characterized by fever and upper respiratory symptoms, accompanied on occasion by vomiting and diarrhea^4^^,^ 5 . Even as some countries reported severe influenza in 2010-11 following the initial pandemic wave,^6^ the data about impact of influenza in the post-pandemic period is limited, especially in the developing regions of the world. In the 2012-2013 season, there was an increased influenza-like illness activity with circulation of both influenza A (H1N1pdm09 and H3N2) and influenza B in countries in the temperate region of Northern Hemisphere^6^ . However, the proportion of A/H1N1pdm09 relative to A/H3N2 increased in some of the European countries, whereas others like the USA and Canada had a predominant circulation of A/H3N2^6^ .It is well established that human influenza viruses evolve rapidly to escape immunity due to prior infections or vaccination^7^ . Because of the possibility for emergence and spread of antigenically drifted variants of A/H1N1pdm09, continued vigilance and monitoring is warranted^6^^,^ 8.

In addition, previous pandemics have shown substantial morbidity and mortality for prolonged periods with a demographic shift in the affected age groups in the post-pandemic periods^9^^,^ 10 ^,^ 11 ;which could indicate either a drift in the virus or a build-up of the immunity in the younger age. Here we report on the recrudescent wave of influenza A/H1N1pdm09 in the winter of 2012-2013 in Kashmir, the temperate northern most region of largely tropical India. This report emphasizes the need for a continued surveillance in order to understand the pattern of circulation and its clinical cost in this region wherefrom the data of influenza circulation has been scarce.

## Methods

The Kashmir province of the state of Jammu and Kashmir is one of the 3 major provinces of the northern Indian state that borders Pakistan, China and Afghanistan. The valley of Kashmir has a temperate climate with respiratory illnesses constituting the bulk of hospital visits during the winter months, either in the form of acute respiratory illnesses or as exacerbations of underlying chronic lung diseases. Sheri-Kashmir Institute of Medical Sciences (SKIMS) is a 650-bed facility in the capital, Srinagar, and is the main tertiary referral centre for respiratory cases for the area^12^. During the winter of 2012-2013, the SKIMS witnessed a surge in visits for acute respiratory illness, many of which required hospitalization. We performed surveillance for outpatients with influenza-like illness (ILI) and in-patients with severe acute respiratory illness (SARI) during the 2010-2011, 2011-2012, and 2012-2013 influenza seasons at SKIMS. We defined ILI as fever of 100^0^F (>37.2 C) accompanied by cough and/or sore throat, whereas SARI was defined as those patients with ILI who also require hospitalization.. Clinical history and examination of the patients was recorded including any history of clustering (two or more cases that were related in time and space, e.g., in a home or workplace) for the entire study period. After clinical data recording, combined throat and nasal swabs were collected in viral transport medium and tested by real-time RT-PCR for influenza viruses using the CDC protocol 13. All influenza A positive samples were further subtyped using primers and probes for A/H1N1pdm09 and A/H3. Virus isolation, haemaggglutination inhibition testing, sequencing and phylogenetic was carried out using standard assay procedures as described previously^14^^,^ 15. Statistical analysis of all data was done using STATA 11 software. Fisher’s exact test and Student’s t-test as appropriate was employed for statistical analysis and differences were considered significant if p<0.05.The study has been approved by the Institute Ethics Committee of SKIMS and informed consent for participation was obtained for all patients.

## Results

We recruited a total of 751 patients meeting the case definitions of ILI (n=545) and SARI (n= 206) from November 26, 2012 to Feb 28, 2013 at SKIMS, Srinagar, India. Of the 751 patients, 184 (24.5%) tested positive for influenza. No major differences were noted in the demographic profile of the influenza positive and negative patients (Table 1). However, influenza positivity was significantly higher in those aged >18yrs (27.1%) compared to those <18 yrs (18.2%; p=0.011) The clinical features among influenza positive cases revealed a significantly higher frequency (p<0.0001) of chills, nasal discharge, sore throat, body ache, and headache and diarrhea in influenza positive patients (Table 1). Further type and subtype analysis of 184 influenza positives revealed that 106 (37.3 %) were influenza A (A/H1N1pdm09 = 105, H3N2= 1) and 78 (42.4%) were influenza B. Longitudinal analysis of surveillance data from January 2011 to February 28, 2013 revealed peaks of influenza circulations during winter months (December-March) with discrete types over the years. Among influenza A viruses, H1N1pdm09 were predominant circulating strains in winter of 2010-2011 and 2012-2013, whereas A/H3N2 was predominant influenza A during winter of 2011-2012. Influenza B activity was observed during peaks of circulation through the entire study period (Figure 1). Taken together, these data suggest a recrudescent wave of A/H1N1pdm09 during the winter of 2012-2013 two year after the pandemic strain first emerged in Srinagar area.

**Weekly trends and seasonality of influenza viruses in Kashmir, India.**
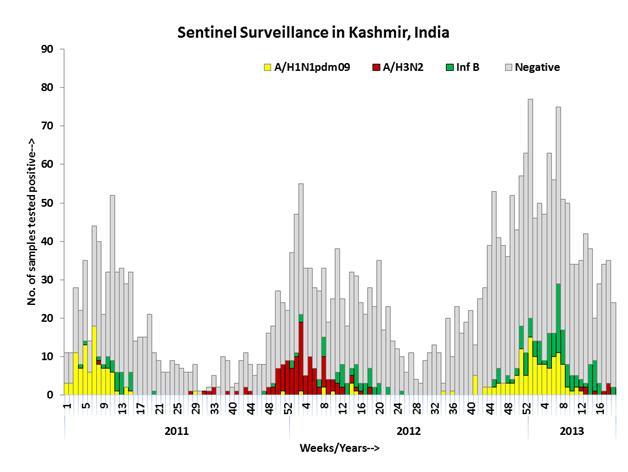
The left axis shows the number of positives for influenza A (yellow bar H1N1pdm09; red bar A/H3N2) and green bar influenza B by epidemiologic weeks for 2011-2013. The total number of cases tested are shown in gray bars for each week from January 2011 to February 28, 2013.

Phylogenetic analysis of a subset of A/H1N1pdm09 (n=6) isolated during 2012-2013 revealed clustering with the original pandemic A/California/07/2009-like (H1N1) pdm09 (Figure 2) Further antigenic analysis of randomly selected A/H1N1pdm09 virus (n=17) by the hemagglutination-inhibition (HI) test using a panel of post-infection ferret antisera revealed antigenic relatedness to A/California/07/2009 (H1N1)pdm09. Additionally, HAI analysis of influenza B (n=10) showed antigenic relatedness to the B/Yamagata lineage.

We next examined the age distribution of the 751 patients studied during the 2012-2013 season Influenza positivity was comparable in children (18%) and those >65 yrs (19%), but significantly higher in those aged >18-65 yrs (28%) (Table 2).

Of the 105 patients from whom A/H1N1pdm09 was recovered, 22.8% (n=24) were aged <18 years, 62% (n=65) were aged 18-60 years whereas 15% (n=16) were aged >60 years.

The positivity rate for A/H1N1pdm09 virus was 11.5% in patients aged 60 age group. Influenza positivity rates for both A/H1N1pdm09 and influenza B were generally higher among outpatients as compared to those hospitalized. However influenza A and Influenza B was observed equally among the hospitalized patients.

Further, we found no major differences in demographic or clinical features among those infected with influenza A/H1N1pdm09 vs influenza B (Table 3). A total of 206 enrolled cases required hospital admission in winter of 2012-2013 and 34 (16.5%) of them tested positive for influenza (A/H1N1pdm09=22; influenza B =11; H3N2=1). Most of the influenza patients who required hospitalization had associated co-morbidities, including chronic obstructive pulmonary disease (COPD, n=13), hypertension (n=19), diabetes (n=8), coronary heart disease (n=4), hypothyroidism (n=3), heart failure (n=3), malignancy (n=3), chronic kidney disease (n=2), and one each with lupus erythematosus, cyanotic heart disease, chronic demyelinating polyneuropathy and panhypopitutarism. Hypoxia was seen in all of the admitted cases (paO2 from 24 to 81(mean 58.12 +13.32) and blood pH ranged from 7.25 to 7.53 (mean + SD 7.38+0.057). Laboratory features among the hospitalized patients included neutrophilic leukocytosis (n=6), lymphopenia (n=9); azotemia (n=9) and elevated LDH (n=16). All except one hospitalized influenza positive patient had radiographic features of pneumonia (right lower zone, 15; right mid zone, 2; left lower zone, 3; bronchopneumonia, 3) or ARDS (n=8). Extensive chest infiltrates were seen in 6 cases on CT imaging. Among the 34 influenza positive hospitalized patients in 2012-2013, all had severe symptoms suggestive of acute pneumonia, acute respiratory distress syndrome or a respiratory failure. Of 34, 28 recovered with routine symptomatic/supportive and antiviral therapy; however, 16 required admission to a high dependency unit (HDU) or an intensive care unit (ICU). Of the 16, 6 patients died with ARDS, progressive respiratory failure and multi-organ dysfunction; 2 had been managed at a different facility for sepsis and were referred after onset of respiratory failure. Of the 6 who died, 4 (ages 1 mo, 11, 17 and 55 yrs) had A/H1N1pdm09; 2 (ages 62 and 75 yrs) had influenza B and died with severe pneumonia (Confusion, Blood Urea, Respiratory rate, Systolic BP, Age>65; CURB scale score >3).The percent of hospitalized SARI patients who tested positive for influenza was comparable in winters of 2010-2011 (8/65, all A/H1N1pdm09; 12%), 2011-2012 (7/57, 6 A/H3N2 and 1 influenza B; 12%), and 2012-2013 (34/206; 16%). However, no fatalities were recorded among the hospitalized SARI patients positive for influenza in the 2010-2011 or 2011-2013 seasons.

## Discussion

We report a recrudescent wave of A/H1N1pdm09 in the temperate region of Kashmir in winter of 2012-2013 (Nov 26, 201 –February 28, 2013), more than 18 months after the previous peak of influenza in the winter of 2010-2011^12^^,^ ^15^. This resurgent A/H1N1pdm09 was associated with frequent hospitalizations and some fatalities. During the winter of 2012-13, other regions of India have also reported circulation of A/H1N1pdm09^16^ .In temperate climates the winter peak of certain ARIs, including infection with influenza is well described^17^^,^ but is not well documented in the tropics^18^^,^ 19. The data from the past three years from this temperate region of India reveals discrete peaks of influenza from December to March in winter season similar to what has been observed in temperate regions of the northern hemisphere^18^^,^ 19 . Unlike North America where the current influenza season was dominated by H3N2 influenza A virus alone^16^ , co-circulation of A/H1N1 pdm09 was also observed in Europe, and tropical countries in Asia region^16^ . Likewise, circulating influenza strains in the Kashmir area in winter of 2012-2013 are predominantly A/H1N1pdm09 with some circulation of influenza B. In the winter of 2012-2013, A/H1N1pdm09 reemerged in Kashmir causing severe illness requiring hospitalizations and fatalities. During the previous winters in 2010-2011 (predominance of A/H1N1pdm09) and 2011-2012 (predominance of A/H3N2), we did encounter influenza cases that required hospitalization, but no influenza-related fatalities were recorded. In the 2012-2013 season, we observed 6 fatalities, four among those infected with A/H1N1pdm09 and two elderly (>65 yrs) with influenza B infection. The antigenic and genetic characteristics of the A/H1N1pdm09 viruses from Kashmir in 2012-2013 are remarkably similar to other circulating strains worldwide. The reasons for the recrudescent wave of H1N1pdm09 in Kashmir area remain unknown, however, it is possible that waning immunity to A/H1N1pdm09 in the population, and exposure of those not affected in the previous pandemic (< 3 yr old; one child was <1 mo old) may be responsible. In a recent study of the clinical, biological and epidemiological characteristics of influenza in the immediate post-pandemic period, the severity of influenza in hospitalized patients during the post-pandemic period was similar to that in the pandemic period^20^. However, radiographic pneumonia was more often diagnosed in patients with A/H1N1pdm09 than those with seasonal A influenza during the pandemic and the post-pandemic periods^20^. Another important observation in the current study was identification of influenza B from patients hospitalized with severe respiratory illness with two recorded fatalities in 2012-2013. Although Influenza B is generally believed to be a rather mild disease^21^^,^ 22 as compared to H3N2, our data reaffirms the potential of Influenza B to be associated with severe and fatal disease. We have recently seen influenza B to be associated with pneumonia (with 4 fatalities) during an outbreak of in a nomadic community in the Himalayan mountain range^22^ ^,^ 23. Severe complications like encephalitis/encephalopathy, influenza-associated myositis and ARDS has also been reported earlier with influenza B in Taiwan^24^.Despite two fatalities linked to influenza B, our data is suggestive of a higher burden of hospitalization, severe complications like pneumonia and ARDS, and mortality from A/H1N1pdm09.

Taken together, our data is suggestive of a recrudescent wave of A/H1N1pdm09 in a temperate climate area of northern India, almost two years after its first appearance in the region 12, associated with severe consequences resulting in significant illness. Continued monitoring of these trends is warranted.

## Figures and Tables

**Figure:**
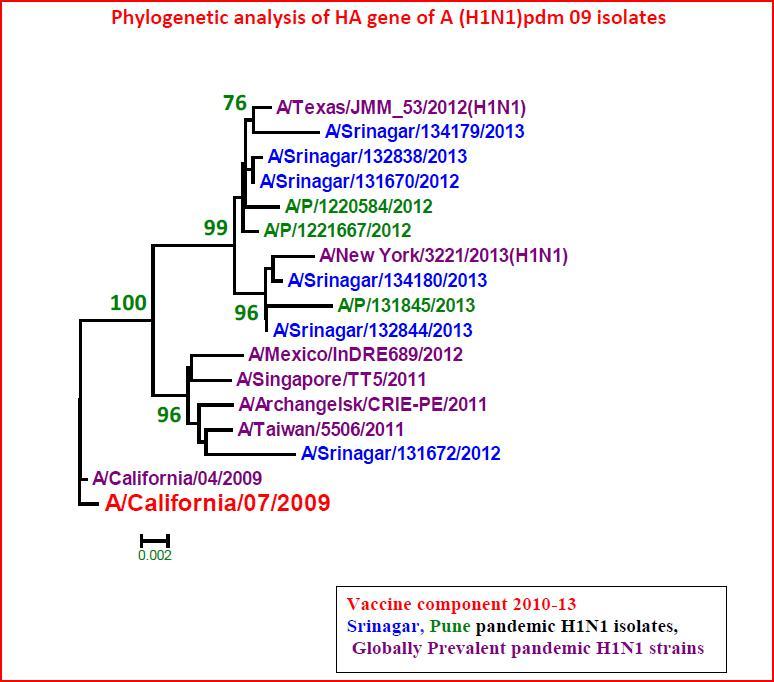
**Phylogenetic analysis of HA gene of A (H1N1)pdm 09 isolates from Kashmir, India.** The representative strains from Srinagar/Kashmir (Blue) and Pune (green) from India and other parts of world (hot pink) and vaccine strain A/California/7/2009 were used to generate the phylogenetic tree by using neighbor-joining method.

## Appendix 1

### Table 1

**Demographic and Clinical features of Influenza positive and negative patients.**

	Influenza positive N(%)	Influenza negative N(%)	p-value
Number	184(24.5)****	567(75.5)	.0001
Males	100(54.3)	301(53)	0.76
Median age ( yrs, range)	35 (1mo-82 yrs)	28.5 (1mo-90 yrs)	0.0716
Median duration of symptoms (days, range)	4 (1-5)	4 (1-6)	
**Clinical features **			
Fever	184 (100)	565 (99.6)	0.72
Chills	174 (94.6)	452 (79.7)	0.0001
Nasal discharge	149 (81)	387 (68.2)	0.001
Ear discharge	2 (1.1)	3 (0.5)	0.60
Cough	177 (96.2)	523 (92.2)	0.06
Sore throat	130 (70.7)	325 (57.3)	0.001
Breathlessness	142 (77.2)	439 (77.4)	0.94
Expectoration	122 (66.3)	337 (59.4)	0.097
Headache	145 (78.8)	353 (62.2)	0.0001
Body ache	149 (81)	385 (67.9)	0.0007
Acute respiratory illness in family	41 (22.3)	105 (18.5)	0.26
* Of total influenza positive, 105 (57%) were with A/H1N1pdm09 and 78 (43%) were Influenza B. No significant difference was observed in clinical presentations between patients with A/H1N1pdm09 or influenza B.			

## Appendix 2

### Table 2

**Age distribution of Influenza positivity among patients with ILI (outpatient clinic attendee) and SARI (hospitalized patients) during winter of 2012-2013 at SKIMS, Srinagar, India.**

	No. tested	No. influenza positive (%)	A/H1N1pdm09 (%)#		Influenza B	
Age Group	ILI	SARI	ILI	SARI
0-<18	219	40 (18.2)	20 (50.0)	5 (12.5)*	13 (32.5)	2(5..0)
>18-65	449	128^^^ (28.5)	60 (46.9)	11 (8.6)*	54 (42.1)	2 (1.6)
>65	83	16 (19.27)	3 (1.9)	6 (37.5)	0 (0)	7 (43.7)**
Total	751	184 (24.5)	83 (45.1)	22 (11.9)	67 (36.4)	11 (6.0)

 (# Percent of total influenza positive

* Fatal cases among hospitalized patients include three children (ages 1 mo, 11 and 17 yrs) and one adult (55 yr) with A/H1N1pdm09

** Fatal cases among hospitalized patients include two adults (62 and 75 yrs) with influenza B; 

^ One specimen was subtyped as A/H3N2)

## Appendix 3

### Table 3

**Comparison of clinical features among Influenza A and Influenza B positive patients:**

	A/H1N1pdm09 n (%)	Influenza B n (%)	p-value
Number	105 (57.4)	78 (42.6)	
Males	52 (49.5)	48 (61.4)	0.11
Median age (yrs, range)	30 (2 mo-80 yrs)	40 ( 2mo -82 yrs)	0.25^*^
Median duration of symptoms (Days, range)	3 (1-4)	4 (1-5)	
**Clinical features**			
Fever	105 (100)	78 (100)	1.00
Chills	100 (95.2)	76 (97.4)	1.00
Nasal discharge	89 (84.8)	60 (77.0)	0.18
Ear discharge	0 (0)	1 (1.2)	-
Cough	103 (98.1)	73 (93.6)	0.12
Sore throat	75 (71.4)	55 (70.5)	0.89
Breathlessness	85 (81.0)	56 (71.8)	0.15
Expectoration	71 (67.7)	50 (64.1)	0.62
Headache	83 (79.0)	61 (78.2)	0.89
Body aches	83 (79.0)	65 (83.3)	0.47
ARI in family	21 (20.0)	20 (25.7)	0.21
Vomiting	11 (10.5)	9 (11.5)	0.82
Diarhhea	3 (2.8)	7 (9.0)	0.07
